# Exploiting animal personality to reduce chronic stress in captive fish populations

**DOI:** 10.3389/fvets.2022.1046205

**Published:** 2022-12-14

**Authors:** Pamela M. Prentice, Thomas M. Houslay, Alastair J. Wilson

**Affiliations:** ^1^Centre for Ecology and Conservation, University of Exeter, Exeter, United Kingdom; ^2^Institute of Aquaculture, University of Stirling, Stirling, United Kingdom; ^3^Ecology and Environment Research Centre, Department of Natural Sciences, Manchester Metropolitan University, Manchester, United Kingdom

**Keywords:** personality fish, stress, welfare, quantitative genetics, selection

## Abstract

Chronic stress is a major source of welfare problems in many captive populations, including fishes. While we have long known that chronic stress effects arise from maladaptive expression of acute stress response pathways, predicting where and when problems will arise is difficult. Here we highlight how insights from animal personality research could be useful in this regard. Since behavior is the first line of organismal defense when challenged by a stressor, assays of shy-bold type personality variation can provide information about individual stress response that is expected to predict susceptibility to chronic stress. Moreover, recent demonstrations that among-individual differences in stress-related physiology and behaviors are underpinned by genetic factors means that selection on behavioral biomarkers could offer a route to genetic improvement of welfare outcomes in captive fish stocks. Here we review the evidence in support of this proposition, identify remaining empirical gaps in our understanding, and set out appropriate criteria to guide development of biomarkers. The article is largely prospective: fundamental research into fish personality shows how behavioral biomarkers *could* be used to achieve welfare gains in captive fish populations. However, translating potential to actual gains will require an interdisciplinary approach that integrates the expertise and viewpoints of researchers working across animal behavior, genetics, and welfare science.

## Introduction

Stress responses are the behavioral and physiological pathways by which animals maintain homeostasis and health when challenged by their environment. From an evolutionary perspective they are considered broadly adaptive (i.e., beneficial for fitness): exposure to acute stressors is part of normal life for wild animals and “stress” should not be equated with “distress”. However, it is also true that chronic stress exposure is a major source of welfare problems in captive animal populations, including fishes (1, 2). For example, chronic stress can cause behavioral changes [e.g., reduced appetite, abnormal swimming patterns (3, 4)], physiological changes [decreased growth, reduced reproductivity, reduced nutritional status (5, 6)], and compromised health [e.g., higher injury rate, compromised immune response (7, 8)].

Given its potential to adversely impact health, stress has long been a focus of research in the biomedical, veterinary and animal sciences (9, 10). Here we define chronic stress following (11) such that an organism can be described as being chronically stressed when there is long-term activation of the hypothalamic-pituitary-adrenal/interrenal (HPA/I) axis caused by unpredictable or uncontrollable stimuli in its environment. Mechanistically, we know that chronic stress effects arise in large part from maladaptive expression of acute stress response pathways. For instance, in humans and other mammals, chronic activation of the hypothalamic-pituitary-adrenal (HPA) axis leads to prolonged elevation of glucocorticoids, which can negatively impact growth, reproduction, and immune function (12). In fishes, prolonged activation of the analogous HPI (hypothalamic-pituitary-intrarenal) axis has similar consequences. However, despite this mechanistic understanding, we remain generally poor at predicting when particular individuals, stocks or species will be negatively impacted. Ideally, there should be a method for high-throughput phenotyping of individuals in order to monitor changes, or—preferably—pre-emptively determine those likely to suffer negative impacts.

Unfortunately, measurement of glucocorticoids [often used as a proxy for stress in vertebrates, although see (13) for a more nuanced overview] comes with a host of practical challenges that make high-throughput phenotyping difficult, even if non-invasive methods are used [see, e.g., (14)]. An alternative, suggested by links between glucocorticoid physiology and behavioral “types” or “styles” [e.g., (15–17)] might be to adopt behavioral testing. Recent technological advances such as video tracking (18) now offer the potential for accurate, semi-automated, and high-throughput behavioral phenotyping. Equally, over the last two decades the emergence and development of “animal personality” as a major research topic in behavioral ecology means we have gained many insights into the causes, consequences and implications of among-individual differences in behavior.

In this article, we aim to highlight how insights from animal personality research could be harnessed to improve welfare of captive animal populations at risk from chronic stress. We focus specifically on potential applications in teleost fishes. This is a reflection of both opportunity and need. On one hand, fishes have been central to animal personality research so there is now a rich literature to draw on. On the other, despite rapid diversification of species used for food aquaculture (19) and their burgeoning use in scientific research (20–22), fish welfare is often viewed as a lower priority than that of—for example—mammals by both the public and the research community [e.g., (23–26); but see (4, 27–30)]. Furthermore, for many fish species now housed in captivity, domestication is recent and ongoing. Recognizing that domestication requires evolutionary adaptation to captive environments over many generations (31–34) provides a potentially useful perspective on the challenge here: if fish are routinely housed in conditions bearing little resemblance to those under which their stress responses evolved (35) it should not surprise us that those responses are ineffective and/or maladaptive in novel captive environments (36). For instance, routine stocking densities used for zebrafish in scientific establishments far exceed those found in wild fish, and can generate social processes that can cause chronic elevation of glucocorticoids (37). Moreover, in most cases behavioral responses—which are the first line of organismal defense when challenged by an acute stressor—are at least partially curtailed in captivity (e.g., a subordinate individual cannot physically relocate to avoid harassment by a dominant one).

Simplistically, two main strategies for improving welfare outcomes in captive fish are used. The first is to try and improve the fit of the captive environment to the fish in the short term. This is done through, for example, enrichment of captive housing condition [e.g., (38–41)]. The second strategy is to try and improve the fit of the fish to the captive environment over the longer term (i.e., generations) through selective breeding strategies. As noted above, domestication involves adaptation to captivity under natural selection (albeit in an “unnatural” environment): some individuals within a stock carry genes better suited to survival and reproduction in the novel, captive environment, and these genes will tend to increase in frequency. Artificial selection can also be applied to target welfare-related outcomes as well as more traditional production traits. In salmonid aquaculture, genetic improvement of production traits *via* well-designed, managed breeding programs is standard, and offers a route to cumulative and permanent gains (32, 42). While selective breeding primarily aims to improve economic efficiency and end point value of finfish species, in many cases these goals are broadly aligned with welfare improvement. This is because many traits targeted for improvement [e.g., growth rate and disease resistance; (32, 43, 44)] are also phenotypes negatively impacted by chronic stress. Simplistically, those individuals (and/or genotypes) with low susceptibility to chronic stress in a given environment should—all else being equal—be the same individuals (and/or genotypes) that have lower disease risk, better growth and higher reproductive success in that same environment.

The goal of the current paper is to highlight how the burgeoning field of “animal personality” might inform selective breeding strategies aimed at improving welfare outcomes. We argue that personality can be viewed as part of the stress response, and that genetically determined behavioral profiles are likely to predict welfare-relevant outcomes in captivity. If so, then integration of behavioral biomarkers into selective breeding programs offers a widely applicable route to genetic improvement of chronic stress resistance. Our argument is largely prospective: personality has become a major sub-field of behavioral ecology over the last decade and studies of fishes have been at the forefront of this [e.g., (45–47)]. We now think the potential for translation to applied contexts is clear, with the highest potential for short-term welfare gains likely to arise in the contexts of housing fish species for scientific research and developing novel species for food aquaculture. However, even in salmonid aquaculture—where genetically informed selection strategies are standard—the utility of behavioral biomarkers remains relatively unexplored [but see (48, 49) for important exceptions]. Moreover, growing recognition of stress-related problems in the pet trade (50, 51) has prompted the suggestion that improved understanding of natural behavior and ecology could greatly benefit welfare in ornamental species (52). That the same suggestion has been made with respect to zebrafish housed in scientific establishments (53) highlights the point that behavioral ecological perspectives should be valuable across aquaculture sectors.

## Personality: What is it and why does it matter?

Before proceeding we briefly outline the concept of *animal personality* for any readers unfamiliar with this area of behavioral ecological research. We also provide a glossary of terms denoted by italic font throughout this article (Box 1). That individual animals differ from each other in behavioral characteristics is intuitive—and perhaps obvious—to anybody that has kept or worked with livestock, laboratory or companion animal species. However, it may be less obvious that this is equally true for wild animals, and seemingly across all taxa examined to date. The field of animal personality is the study of these differences, with researchers adopting standardized behavioral tests and quantitative measures so that hypotheses about the causes and consequences of behavioral differences can be formally tested.

Box 1 **Glossary**

*Brief definition, and a reference to further reading where appropriate*.

*Animal personality:* Consistent or repeatable differences in behavior among individuals across time and/or contexts. This among-individual variation is attributable to the combined influences of genetic and environmental effects that permanently affect the phenotype of an individual. Typically quantified as *repeatability*, and often used as an informal indicator of the upper limit of the *heritability* of behavior. Commonly studied personality traits include aggressiveness, activity, boldness, exploration, and sociability [see (54)].

*Breeding value*: The deviation (or effect) of each genotype from the population mean.

*Correlational selection:* selection for optimal trait combinations [see (55)].

*Genetic correlation:* The correlation of trait breeding values. Can be used to describe the association between different traits on the same individual or between the same traits on different individuals in different environments (where an “imperfect” correlation indicates genotype-by-environment interaction). Genetic correlations are largely caused by pleiotropy (when one gene influences multiple traits) but can also be caused by linkage disequilibrium (non-random association of alleles at different loci within a population).

*Heritability:* The proportion of phenotypic variance that is due to additive genetic effects [see (56)].

*Integration:* Correlation structure among suites of traits. Can be studied at levels including phenotypic, among-individual, and genetic.

*Jingle-jangle fallacies:* Pervasive and misleading over- or under-labeling of traits. A “jingle” fallacy occurs when a single trait label inadvertently describes two functionally different traits measured with different tests. A “jangle” fallacy occurs when two different labels measure the same trait [see (57)].

*Latent variable/axis of variation:* A variable that is not directly observed but rather inferred from a model of observed data.

*Phenotypic plasticity:* Expression of different phenotypes by a single genotype, often in response to environmental variability [see (58)].

*Repeatability:* The proportion of phenotypic variance due to differences among individuals [see (59)].

*Stress coping styles:* Characterizes the behavioral and physiological responses of individuals to a stressful situation. Distinct styles, or the extremes of a continuous axis, are often labeled as “proactive” (exhibiting strong responses to stimuli) and “reactive” (more passive responses) [see (15)].

**Figure 1:**
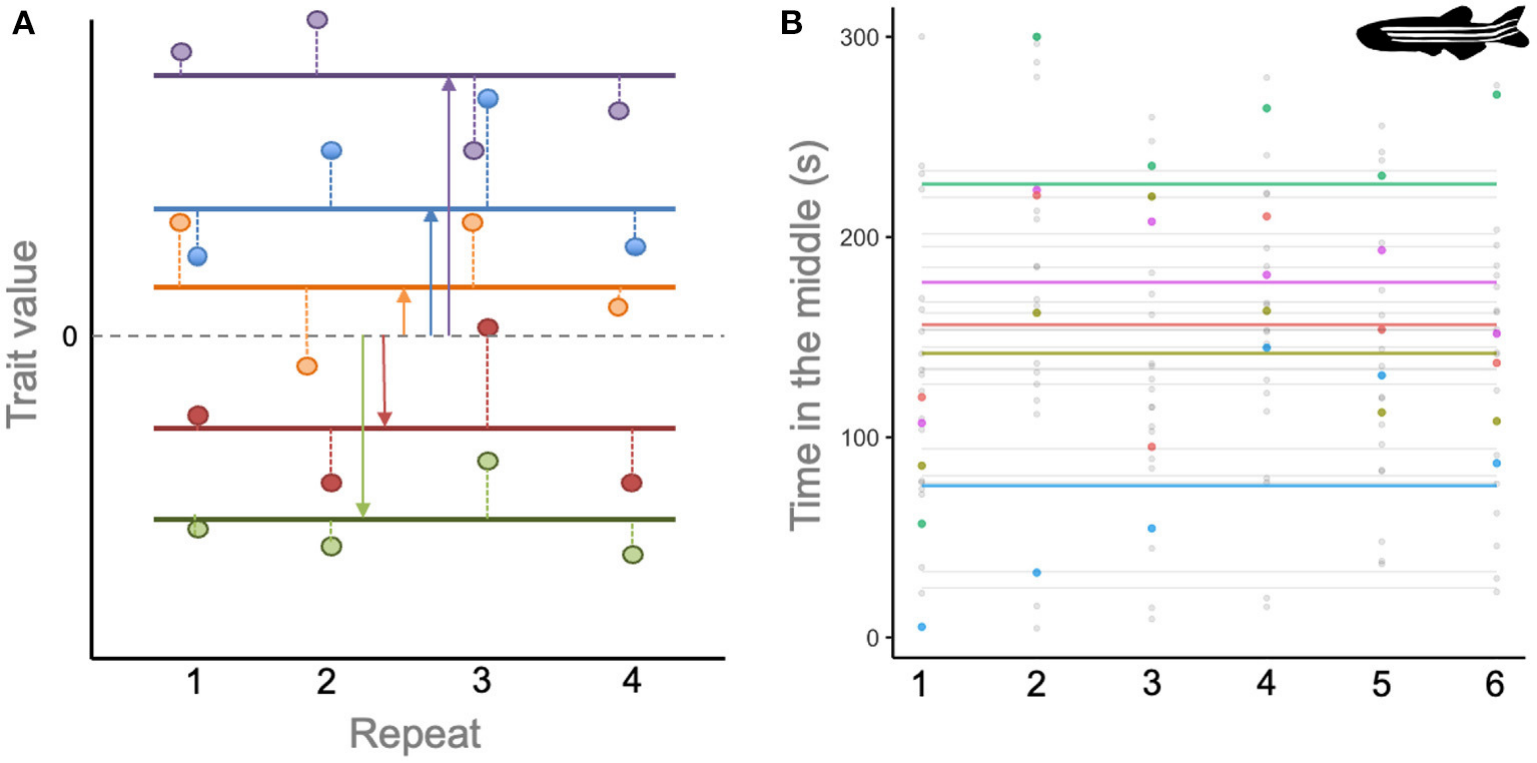
**(A)** Illustration of how repeated observations (points) on different individuals (colors) can be used to partition among- from within-individual variation in a population. The horizontal dashed line shows the population mean, and horizontal solid lines the mean for each individual. Solid vertical lines show the extent to which individuals differ (on average) from the population mean. Dashed vertical lines show how an individual's observations vary from their own average value (i.e., the within-individual or residual variance). **(B)** Observations (points) and individual-specific means (horizontal lines) for duration of time in the middle (inner) zone for 24 zebrafish (*Danio rerio*) observed over six open field trials. Five representative individuals have been colored to highlight their means and observed values. The adjusted repeatability of this behavior within the study was estimated at 0.56 from a mixed model analysis. Data redrawn from (60) with permission. *Danio rerio* silhouette by Josefine Bohr Brask and used under Creative Commons Licence BY-NC-SA 3.0 (https://creativecommons.org/licenses/by-nc-sa/3.0/).

Despite attempts to standardize terminology [e.g., (61)], the rapid emergence of this field over the last two decades has resulted in plurality of definitions, abundant semantic arguments, and many *jingle-jangle fallacies* (57). For present purposes, we hope to avoid these issues by focusing on two points of consensus. First, just as in human psychology, animal personality traits are conceptualized as *latent axes of variation* that underpin observed behaviors. So if a population is hypothesized to contain individuals differing in traits such as boldness, aggressiveness or sociability then these aspects of personality are typically investigated *via* measurable proxies. For instance, if a population of fish is characterized by variation in aggressiveness, we would expect some individuals to rapidly attack a mirror stimulus more than others, and/or to spend more time displaying to a rival, and/or to chase tank mates more frequently (62). Second, personality studies generally target an understanding of differences in behavior among-individuals that are consistent (or repeatable) across time and/or context. This is distinct from within-individual variation that arises from *phenotypic plasticity* of behavior in response to extrinsic environment, social context, physiological state, or motivation. If within-individual variation is high, then any single observation will contain little information about that animal's underlying personality, and so whether or not it differs from any other individual. Conversely, if the behavior of individually identifiable animals is observed repeatedly, then the among- and within-individual components of variation can be statistically partitioned (Figure 1). Thus, repeated measures designs are the usual hallmark of animal personality studies (54, 63, 64).

The importance of distinguishing among- from within-individual variation in personality research stems from the evolutionary principles and adaptive framework that underpins behavioral ecology. Differences among-individuals are a pre-requisite for natural selection, which occurs when differences in phenotype cause differences in fitness (65), and also for a response to selection. More formally, in quantitative genetic theory the *repeatability* (R, the proportion of variance explained by individual identity) sets an upper limit for its *heritability* (*h*^2^, the proportion of trait variance explained by additive genetic effects). In turn, the heritability determines the fidelity with which selected phenotypes are transmitted from parents and offspring and so the rate of evolution for a trait under selection (56).

## Personality as a component of the stress response

Studies targeting physiology dominate the empirical literature on stress response. Indeed an overly narrow, albeit pragmatic, focus on glucocorticoid physiology means that “stress” and cortisol are sometimes treated as synonymous [but see (13)]. However, conceptual models such as *stress coping style (SCS)* (15) explicitly recognize the importance of behavior. SCS proposes functional *integration* of neuroendocrine and physiological pathways with individual behavioral profiles. Under SCS a “reactive” stress response is characterized by behavioral immobility (e.g., freezing) coupled to lower glucocorticoid response, while “proactive” types are predicted to show more active “fight-or-flight” behaviors and higher glucocorticoid levels (15). The behavioral aspect of SCS is equivalent to the personality concept used in behavioral ecology, and at least analogous to shy-bold type personality variation (66). While boldness is usually defined as an axis of variation in behavioral responses to perceived risk (54), the specific testing paradigms and behavioral measures used in fish models have equal validity for assaying acute stress response. These include, for example, assaying time to resume feeding or leave a shelter after a simulated predation event (e.g., net chase), quantification of neophobic responses to novel objects, and measurement of thigmotaxis (wall-hugging). Freezing and/or “flight-type” swimming behavior after being placed in an open field arena have also been used [e.g., (67, 68)] highlighting that variation can equally be characterized by extracting movement parameters (time stationary, step length, turning angle, and burst frequency) from tracking data (69). Verbal models are, of course, open to subjective interpretation; whether studies of boldness in fish reveal among-individual variation more consistent with differences in the “style” as opposed to the “magnitude” of a behavioral stress response is a fine distinction that may be difficult to resolve in practice [see (67) for an attempt to do so in guppies]. For current purposes, however, what to call personality variation revealed by exposure to acute stress stimuli is of lesser importance than determining whether it is correlated with stress physiology, and/or predictive of welfare outcomes under chronic stress exposure.

There is now abundant evidence that fish populations harbor high levels of shy-bold type personality variation. This conclusion is taxonomically general, although a number of small teleost model species have contributed disproportionately to the literature, notably guppies and sticklebacks in behavioral ecology, e.g., guppies (70), sticklebacks; (71, 72), rainbow trout in aquaculture [e.g., (73)], and zebrafish in biomedical research (74, 75). Moreover, quantitative genetic analyses across a range of fish species have shown that among-individual differences are at least partially explained by heritable genetic effects (76–78). Increasingly the goal for behavioral ecologists studying fish personality is to go beyond simply characterizing patterns of repeatable and/or heritable variation, by further exploring the ecological and functional relevance. For instance, how stable is personality across different stress contexts (47) or temperatures (79)? Are particular personalities associated with parasite susceptibility (80) or pollutant exposure (81)? Do bolder individuals disperse more (82)? Are some personality types more attractive to potential mates (83) or favored by selection because they yield greater reproductive success (84)? A general criticism of the personality approach from within behavioral ecology is that reliance of simple standardized behavioral assays, often conducted under laboratory conditions, might tell us less about the structure and function of among-individual differences in wild populations than we hope. This criticism has validity, but also a useful corollary: if studies of shy-bold variation in captive fish populations may sometimes lack ecological relevance, they typically involve manipulations and presentation of stressor stimuli that are very relevant to life in captivity.

## Integration of personality and stress physiology in fishes

We now know that personality differences are ubiquitous in fishes. At the same time, longitudinal, repeated measures approaches have become more commonly used in fish physiological studies, revealing that among-individual differences in stress-physiology are common [e.g., (47, 85, 86)]. However, to what extent are personality and physiological traits *integrated* as widely predicted (9, 10, 36, 87)? At this juncture it is worth noting that the concept of “integration” can be understood from both proximate (mechanistic) and ultimate (evolutionary) perspectives. In the former sense, phenotypic correlations between neuro-physiological and behavioral traits associated with acute stress response are clearly expected. For example, activation of the sympathetic nervous system is causal to fight-or-flight responses, while glucocorticoids released by the HPA(I) axis are known to impact behavior extensively (88, 89). However, from an evolutionary perspective, we are primarily interested in the extent to which genetic effects underpin correlations among traits as this determines their potential to evolve independently. Natural selection does not act on single traits in isolation but rather on multivariate phenotypes (90–92). Moreover, if there is no single optimal combination of trait values, then *correlational selection* can favor multiple combinations that yield relatively high fitness, maintaining variation and—over time—leading to the emergence of among-trait correlations (93). Strictly speaking, this argument predicts the emergence of *genetic correlations* among traits but—for reasons outlined above with respect to single traits—these should be reflected in among-individual correlations (as opposed to within-individual correlations driven by plasticity).

In fishes, studies of stress-related physiology-behavior correlations remain limited, and more direct tests of the genetic and among-individual correlations predicted by evolutionary arguments for integration are needed. Support for integration comes primarily from studies of commercially important aquaculture species (94) including Senegalese sole [*Solea senegalensis*; (95)], olive flounder [*Paralichthys olivaceus*; (96)] and mulloway [*Argyosomus japonicus*; (97)], and there is additional evidence from ecological models such as sticklebacks (98, 99) and Poeciliids [e.g., (66)]. Broadly, the emergent pattern is consistent with integration of behavior and physiological stress response traits in fishes, though not all relationships match specific predictions from verbal models such as SCS [see e.g., (66)]. In other words, populations do harbor among-individual variation in multivariate stress response and there is correlation structure among behavioral and endocrine traits. However, across populations and species it is much less clear whether, for example, high cortisol responses to acute stressors are always associated with behavioral trait characteristics described as “shy” (in the personality literature) or “reactive” (under the SCS model). In addition, more evidence is required to determine whether high cortisol responses to acute stressors do predict chronic activation of the HPA/I axis—and whether there are other biomarkers that may provide a more robust signature of chronic stress in fish [e.g., (100)].

**Figure 2:**
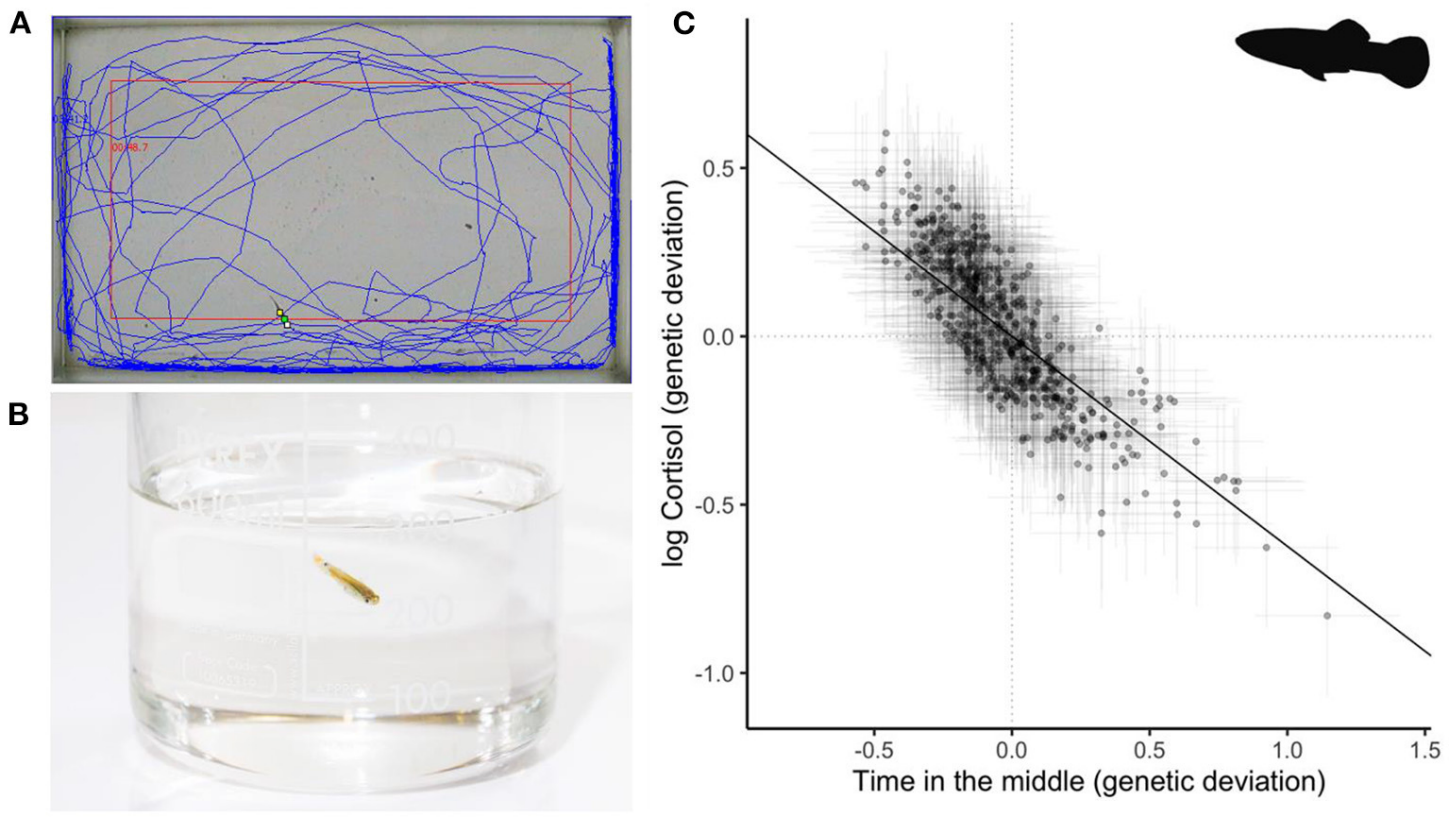
Example from a recent study of stress-related behavior and glucocorticoid response in Trinidadian guppies (*Poecilia reticulata*) by Houslay et al. (67). **(A)** Individuals from a pedigreed population were observed repeatedly in open field trials (OFTs). Blue track shows a summary of individual movement over the trial period; red rectangle indicates the overlaid zoning (inner or middle zone vs. outer zone). **(B)** Individuals were also measured repeatedly for their free circulating cortisol response to a mild stressor of handling and isolation. **(C)** Points show (predicted) genetic deviations from the overall means for time in the middle (x axis) and ln-transformed cortisol (y axis) of this pedigreed population. Horizontal and vertical error bars around each point show standard errors on these estimates. The black line shows the regression line, calculated as cov(y,x)/var(x) from the genetic variance-covariance matrix estimated in a multivariate animal model. Data redrawn from Houslay et al. (67) with permission. Photograph by T. Houslay. *P. reticulata* silhouette by Ian Quigley used under Creative Commons Licence BY-NC-SA 3.0 (https://creativecommons.org/licenses/by-nc-sa/3.0/).

A series of studies on rainbow trout (*Onchorynchus mykiss*) have proven particularly influential because they harness the power of artificial selection to demonstrate integration at the quantitative genetic level. Replicate lines of trout selected for high and low plasma cortisol response to a confinement stress test (101) showed correlated changes in stress-related behavior (16, 102–105). Although this clearly evidences genetic correlation structure between physiology and behavior, some later results from this series of studies appear inconsistent and/or context-dependent complicating the interpretation somewhat (106–110). More recently, using a quantitative genetic breeding design coupled to repeated measures of behavior and physiology in wild-type guppies (*Poecilia reticulata*) (67), showed evidence of genetic correlation structure between stress-related behavioral traits (e.g., thigmotaxis and freezing) expressed in open field trials (OFT) and free circulating cortisol levels produced in response to an isolation and confinement stressor (Figure 2). This finding means that artificial selection on personality assayed by OFT would be expected to result in correlated evolution of cortisol physiology.

## Can we use personality to predict welfare outcomes?

Accepting that personality forms part of an integrated acute stress response (47, 67), and that differences in acute stress response are linked to differences in chronic stress susceptibility (27, 29), it follows that at least some aspects of personality should be useful in predicting welfare outcomes in captivity. Unfortunately, very few studies have tested this proposition directly, which is perhaps a reflection of poor communication between fundamental biologists studying fish behavioral ecology, and more applied researchers working with aquaculture populations. In fact the importance of behavioral variation is widely recognized in aquaculture as behavioral signs nearly always provide the primary warning of emerging health and welfare problems. The recent development of automated tools to detect deviations from normal behavior (111) should therefore prove a valuable addition to the armory of facility staff and managers charged. However, at present fish behavior is only used to identify problems that have arisen, not to predict—and so avoid—future problems.

Conversely, for more than a decade behavioral ecologists have been very interested in the extent to which individual personality predicts evolutionary “fitness” outcomes (112). Although these include variables such as mortality risk, reproductive output and disease susceptibility that are also relevant as welfare indicators in veterinary and animal science, the ultimate goal is usually to understand the fitness consequences of personality variation in wild populations. The emergent picture from this field is complex; personality traits are often correlated with fitness measures (82, 113), but counter examples are easy to find in the literature [e.g., see (112)], and relationships can be state and/or context dependent [e.g., (114, 115)]. In a recent multi-taxa meta-analysis of shy-bold personality variation, which we have argued is closely related to the acute stress response, Moiron et al. found that bolder personalities tend to have higher survival (116). Interestingly, however, this pattern was driven by studies of wild animal populations and no relationship was detected in the subset of studies on captive laboratory populations. Clearly there is need for caution when generalizing across contexts: wild populations experience some agents of mortality that are neither applicable to captive scenarios nor linked to chronic stress. For instance, in guppies (*Poecilia reticulata*) bolder individuals have greater survival in the presence of predators (117). However, this is not because bold fish are better able to tolerate the chronic stress of living under high predation threat, but because they are better able to avoid the rather more acute threat of actually being eaten.

Although data remain limited, chronic stress is implicated in at least some cases of personality-fitness correlations in captive fishes. This has perhaps been best evidenced where animals are group housed and competitive interactions are a source of social stress. For example, in a study of captive Sheepshead swordtail (*Xiphophorus birchmanni*), individuals that showed lower activity levels in open field trials also tended to have behaviorally dominant personalities, faster growth and longer lifespan (77). Moreover, the presence of behaviorally dominant aggressive fish reduced the growth rates, and probably lifespans, of subordinate tank mates (118). This illustrates an important consideration, and potential complication for managers: those individuals (and genotypes) best able to tolerate chronic stress may sometimes be the same individuals (and genotypes) that are a major source of stress for conspecifics (119). This may well be the case if it turns out that bolder personalities are usually more resistant to chronic stress, since personality research has shown that—counter to the example provided above—boldness and aggressiveness are often positively correlated among-individuals (71, 120, 121). Lessons from salmonid aquaculture are also informative here. Successful selection for fast growth has led to correlated increases in boldness, risk taking and aggression in farmed fish, creating a social environment that is detrimental to the welfare of more risk averse, less aggressive individuals (94, 122). Quantitative genetic models developed for application to social interactions in pigs and poultry offer practical ways forward (123, 124). A full treatment of this topic is beyond the scope of this article, but a key lesson is that in selecting to improve welfare outcomes, it will sometimes be necessary to jointly consider—and balance—a genotype's susceptibility to chronic stress with its propensity to cause chronic stress.

## What makes a good biomarker for improving chronic stress resistance?

We have argued that captive populations of fishes ubiquitously harbor among-individual differences in personality that is underpinned, at least in part, by genetic factors. We have also argued that personality can be understood as one component of an integrated stress response, and that (genetic) variation in the acute stress response is expected to predict susceptibility to adverse effects of chronic stress. We now need more empirical studies, across a range of species and contexts, to verify the predicted associations between individual personality and welfare-relevant outcomes. If the goal is genetic improvement of welfare outcomes, these studies will need to employ quantitative genetic approaches to assess whether, and to what extent, targeted indicators are heritable and evolvable. This of course means choosing and measuring appropriate welfare indicators, which is challenging in its own right (8, 125). In practice three broad approaches to defining fish welfare are common (126, 127): “feelings-based” approaches that focus on subjective mental state and aim for animals to be free from negative experiences such as pain, fear or distress; “nature-based” approaches that seek to ensure expression of natural behaviors; and “function-based” approaches that emphasize the ability to adapt to the present environment such that animals are in good health and capable of homeostasis. In practical terms, the third approach will generally be easiest, as many functional indicators such as growth, mortality, disease incidence and reproductive performance are already monitored in fish populations.

Assuming genetic potential for improvement is present, selection on behavioral biomarkers offers several advantages—at least in principle—relative to alternative phenotypic targets. First, it would allow selection for chronic stress resistance without having to impose chronic stress and cause adverse effects. Importantly, while chronic stress exposure is the largest threat to welfare in intensive fish husbandry systems (4, 125), there is no expectation that occasional acute stressor exposure causes lasting harm to health. Indeed adverse effects have not been generally detectable using standard assays of personality variation. Second, increasingly widespread use of video tracking (18, 128) means high-throughput phenotyping is easier and cheaper for behavior than for stress-related physiological traits. Low cost camera setups exist (129) and robust open source tracking software is now available [e.g., (130)], reducing entry costs and meaning there should be few ongoing consumables once phenotyping systems are established. Set against these advantages are some very real challenges in developing appropriate behavioral biomarkers. We think these challenges will be best met by combining expertise of behavioral ecologists, quantitative geneticists, veterinary and welfare scientists, and—perhaps most critically of all—the facility and technical staff working to promote welfare on a daily basis. Simplistically, a useful biomarker must allow accurate selection of individuals with genotypes conferring high chronic stress resistance. However, it must also be practicable, allowing low cost high throughput phenotyping that is easy to integrate with existing husbandry practices. Inevitably, these two requirements will sometimes trade-off and compromise will be needed. Nonetheless, it is possible—and we hope useful—to identify the key features of an ideal behavioral biomarker:

(i) *Heritable and genetically correlated with target welfare indicator(s)*

These criteria must be met if selection on a biomarker is to produce an evolutionary response. All else being equal, the more heritable the biomarker is—and the stronger the genetic correlation with welfare indicator—the more accurate the selection and the faster the genetic improvement in welfare. The expected rate of improvement also depends on the intensity of selection, and the level at which selection on phenotype is performed. Behavioral ecologists typically think of (natural) selection acting at the among-individual level, but among-family or within-family artificial selection schemes are often used in aquaculture for practical reasons (131). Relative to selecting on phenotype alone, the accuracy of selection can be greatly improved by use of genomic data (32), and/or pedigree-based methods of predicting genetic merit. We acknowledge that, in principle, genomic selection to improve welfare traits is the “gold standard” and may have little to gain from integration of behavioral data. However, in practice associated costs mean genomic selection is not generally accessible beyond high value commercial species. Moreover, we note that even relatively low accuracy selection may produce very tangible gains. For example, many fish species used in scientific research (e.g., medaka, zebrafish, and guppies) are maintained at high effective population sizes (meaning selection gains are less likely to be lost by drift) and have short generation times (meaning modest gains per generation can accumulate more rapidly over annual time scales).

(ii) *Generalizable*—*across environments, populations and species*

Ideally, a behavioral predictor developed and validated in one population would also be a useful tool to improve welfare in others. The extent to which this may or may not turn out to be the case is unknown. Although the behavioral ecology literature has often emphasized the idea of a discrete set of latent personality traits or axes that are conserved across taxa, many (including ourselves) consider this more as an organizing framework than an empirical reality. Here we have focused on shy-bold type variation as being analogous to the behavioral component of stress response, but we do not know if, for example, shy or bold individuals will be generally more robust to chronic stress exposure. Moreover, we know that, even if subjected to a single standardized assay in a controlled environment, the amount and/or structure of behavioral variation among-individuals is likely to differ among populations and species [e.g., (60)]. We also know that among-individual variation can be a function of environmental context (132), and exposure to stressors can alter behavior-physiology relationships (133). In this context the question is not really whether a biomarker that is optimized for one scenario will be similarly ideal for all other—it will not be. However, the hope is that at least some limited generality emerges such that tools developed in one population provide useful starting points for improving welfare in others.

(iii) *Measurable in early life*

**Figure 3:**
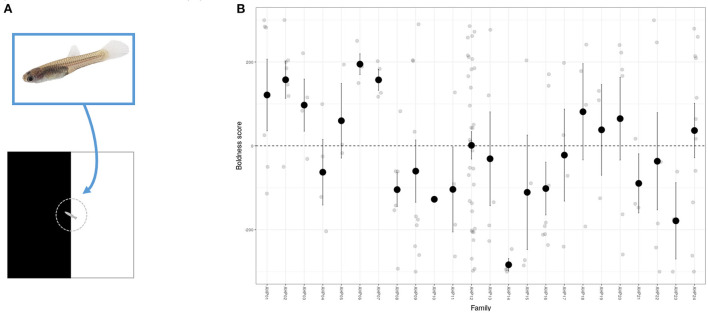
Among-family variation in boldness in juvenile guppies. **(A)** Using a simple scototaxis assay for anxiety like behavior juvenile guppies are individually transferred to cylinder and then released into a 5 × 5 cm arena equally divided into black and white sections. A camera placed above allows video tracking and measurement of boldness. Here we define a simple boldness score from a 300 s observation period as time over light background –time over dark background (such that higher values indicate greater preference for light background). **(B)** Individual observations (grey points) on 149 fish from 24 families (of 1–33 offspring, mean brood size = 12) aged 2–14 days (with a of 6 days). Fish consistently prefer the dark background (negative boldness scores), with dashed line at boldness = 0 corresponds to no preference. However, mean behavior also varies significantly among-families (black points and associated error bars depict family means with 95% CI; tested conditional on age, *F*_23,124_ = 1.71, *P* = 0.034) consistent with genetic variation for boldness.

Shy-bold type variation can readily be detected in fishes at young ages. An inability to tag small individuals for identification has limited the ability to estimate among-individual variation directly but in open field trials differences in mean behavior have been found among strains of zebrafish embryos at 12 days post-fertilization (134), and among guppy families aged between 35 and 55 days (78) (Figure 3). This means selection on behavioral biomarkers could be conducted in early life, reducing the need to raise larger number of fish and decreasing associated financial costs. Although individual personality differences can be broadly stable across ontogeny in some cases (46) this may not be generally true (135), which raises two practical considerations. First, if components of variance are age (or life-stage) specific then a chosen biomarker may be more repeatable (and/or heritable) at some ontogenetic stages than others. This means the ability to usefully discriminate among individuals may depend on the age at which behavioral assays are performed. Second, if individuals and genotypes change rankings over ontogeny (e.g., some genotypes predisposing to boldness in early life but shyness later, and *vice versa*) then it is possible that the sign—as well as magnitude—of the genetic correlation between the target welfare indicator could actually depend on age. Although this would be an extreme scenario it highlights that—while the ability to select early in life is useful—the extent to which early life behavioral phenotype effectively predicts later life stress resistance needs to be checked.

(iv) *Simple, fast, and cheap to measure*

Behavioral ecologists deploy various simple behavioral assays—either singly or in combination with each other—to investigate shy-bold type personality variation in fishes (136, 137). These commonly include open field tests, emergence tests (in which willingness to leave a shelter and enter a potentially risky environment is assayed), novel object trials (in which willingness to investigate an unfamiliar object is tested). Tests of responses to simulated predation attempts are also used in an ecological context (47). Added to this are protocols developed by biomedical researchers to assay “anxiety-like behavior” in fish [e.g., by testing dark/light preference or scototaxis; (138)]. In general, all tests are simple and quick by design, and most allow data extraction and processing that can be at least semi-automated (e.g., by using video tracking software coupled to cameras).

For current purposes the “best” assay will be one that yields predictive biomarkers of welfare outcomes in a target stock, but is also scalable to suit the needs and resources of a particular facility. In exploring options to achieve this, several points are worth bearing in mind. First, behavioral data should, where possible be collected on continuous rather than binary or categorical scales. So it is better, for example, to record the time taken for an individual to emerge from a shelter rather than simply whether or not it did emerge. This is because it contains more information and is almost always easier to analyze. Second, recording multiple behavioral proxies from a single assay can be very helpful. For example, in guppies tracked in an open field arena, high distance swum over a short observation period can be indicative of systematic exploration by putatively unstressed fish, or of a “flight-type” stress behavior. These possibilities are readily distinguished by jointly extracting multiple behaviors from the tracking data since, for example high activity coupled to thigmotaxis is diagnostic of a flight response in this species (67). Multivariate behavioral data can be reduced to a scalar measure prior to analysis using simple dimensionality reduction techniques like principal component analysis (PCA). However, in practice this may not be desirable since it involves loss of information and a better strategy will usually be to regress multiple predictor behaviors on the targeted welfare indicator. The partial regression coefficients of each trait on the welfare indicator could then be used as weightings in a linear selection index (56). This process is directly analogous to the standard strategy used by evolutionary ecologists in which multiple regression of traits on fitness is used to estimate the vector of linear natural selection (90). Where repeat measures and/or pedigree data are available then established mixed model strategies to estimate these partial regressions at the among-individual and additive genetic levels are already readily available (139, 140). The key idea here is that while the simplicity of using just one behavior (or composite trait defined using PCA) is appealing, it is generally the case that using more information to inform selection will yield better results.

Third, and finally, in adopting methods from animal personality research we must avoid the trap of being constrained by its conventions. Following the definition of personality as behavioral differences that are consistent across time and context, many behavioral ecologists take the view that multiple observations on individuals are essential to work on this topic [see (141) for a balanced discussion on this]. Since absolute sampling effort is limited (e.g., by researcher time) power analyses are particularly useful to balance the inevitable trade-off between sampling more individuals and obtaining more repeats per individuals [e.g., (142)]. However, in the present context, the primary value of multiple observations is practical not conceptual; repeated observations of a biomarker allow more accurate selection because averaging across multiple observations within an individual reduces the signal of measurement error and short term plasticity. The degree of benefit depends on the number of repeats—more is always better but the returns are diminishing. It also depends on the trait's repeatability—less is gained by obtaining multiple observations on a trait known to have high R (56). However, repeated measures designs usually require individuals to be tagged for identification purposes which imposes costs. They also requires repeated handling of animals that could actually cause adverse effects. Thus, while more accurate selection is desirable, obtaining repeated measures on individual fish is not actually essential if practical constraints make it prohibitive.

## Conclusion

Rapid increases in aquaculture production for food, and in the use of fish models in scientific research mean that more fish than ever are being housed in captivity. Consequently there is a need to develop and implement strategies that protect welfare by reducing adverse effects of chronic stress. Here we have used insights from the study of “animal personality” to show how, and why, selection on behavioral biomarkers could be used to reduce chronic stress susceptibility and improve welfare outcomes across cultured fish species. This potential exists because: (1) captive populations of fishes harbor among-individual differences in personality that are underpinned by genetic factors; (2) personality can be understood as one component of an integrated acute stress response; and (3) differences in acute stress response phenotypes will predict differences in susceptibility to adverse effects under chronic stressor exposure. With these three conditions met then (4) integrating behavioral biomarkers into selective breeding programs offers a route to genetic improvement of chronic stress resistance. In seeking to stimulate discussion among researchers with different areas of expertise we have focused our arguments on broad principles and acknowledge that our treatment of some technical topics (e.g., design of selection strategy) is somewhat superficial—but we hope accessible—as a consequence. We also acknowledge that further empirical studies are needed and, in particular, estimates of the quantitative genetic (co)variance structure between shy-bold type behaviors and defined welfare indicators would be very valuable. More widespread collaboration between behavioral ecologists and geneticists will allow us to better determine the potential for welfare gains across species and populations. However, realizing those gains will then require input and technical expertise from aquarists and facility staff to develop phenotyping platforms and selection strategies that are not just effective, but are also practical and cost effective to embed into breeding programs.

## Data availability statement

This article includes some secondary presentation of published data. Data associated with the original contributions are included in the relevant articles/supplementary material, but any further inquiries can be directed to the corresponding author.

## Ethics statement

The manuscript includes some secondary data use of behavioral data collected on fish. This work was approved by the University of Exeter Animal Welfare and Ethical Review Board as well as by the UK Home Office.

## Author contributions

All authors listed have made a substantial, direct, and intellectual contribution to the work and approved it for publication.
